# Soluble Fibrin Monomer Complex and D-Dimer Concentrations Between Patients at Low and High Risk of Venous Thromboembolism Before Delivery According to RCOG Score Assessment: An Observational Study Among 100 Third-Trimester Vietnamese Pregnancies

**DOI:** 10.3390/jcm14051399

**Published:** 2025-02-20

**Authors:** Anh Dinh Bao Vuong, Ngoc Hai Tran, Thanh Hai Pham, Hoai An Minh Le, Phuc Nhon Nguyen

**Affiliations:** 1 Department of High-Risk Pregnancy, Tu Du Hospital, Ho Chi Minh City 71012, Vietnam; baoanh1357@gmail.com; 2 Clinical Research Center (CRC), Tu Du Hospital, Ho Chi Minh City 71012, Vietnam; bsngochai.bvtudu@gmail.com (N.H.T.); haiphamtd@gmail.com (T.H.P.); 3 Laboratory Department, Tu Du Hospital, Ho Chi Minh City 71012, Vietnam; leminhhoaian@gmail.com

**Keywords:** D-dimer, maternal mortality, high-risk pregnancy, soluble fibrin monomer complex, RCOG, venous thromboembolism

## Abstract

**Background/Objectives:** Venous thromboembolism (VTE) is related to maternal mortality, especially after the coronavirus disease (COVID-19) pandemic. The Royal College of Obstetricians and Gynecologists (RCOG) guidelines’ risk assessment score has been established to reduce thrombotic complications during pregnancy. Recently, it was found that the soluble fibrin monomer complex (SFMC) could be an alternative to D-dimer (DD), which has been used to assess the risk of VTE. This study aims to reveal the difference between FM and DD concentrations in low- and high-VTE-risk groups according to the RCOG’s guidelines. **Method:** This observational study was conducted at the Department of High-Risk Pregnancy, Tu Du Hospital, Vietnam between August 2023 and April 2024. This study enrolled 100 pregnant women beyond 28 weeks of gestation at low risk (≤2 points) and high risk (≥3 points) of VTE assessment following the RCOG guidelines’ score. Blood samples were collected for the SFMC and DD tests before delivery. Statistical tests were used to compare the difference in SFMC and DD concentrations between the two groups. A *p*-value < 0.05 is considered statistically significant. **Results:** We found no significant difference in DD and SFMC tests between low and high VTE risk (1.61 [1.30–2.30] vs. 1.51 [0.91–2.13]; 5.00 [1.36–9.78] vs. 3.74 [1.28–14.63], respectively; *p* > 0.05). The length of hospital stay in the high-risk group is longer than that of the low-risk group and involves postpartum infection. In addition, we found no significant correlation between the gestational age and SFMC or DD concentration. However, a moderate positive correlation between the two tests was found. Similarly, no significant correlation between the VTE score and SFMC or DD concentration was found in the present study. **Conclusions:** The soluble fibrin monomer complex and D-dimer tests are not significantly different between low-risk and high-risk groups determined through VTE evaluation before delivery according to the RCOG guidelines. The fibrin-linked tests need to be individualized and applied among pregnant women with higher scores of VTE risk based on maternal and pregnancy characteristics during antenatal care. Further studies with a larger number of participants are required to strengthen the findings.

## 1. Introduction

A globally worsening issue, hypercoagulation leads to severe complications during pregnancy and increases morbidity and mortality for pregnant women. This serious complication can involve venous thromboembolism (VTE), deep vein thrombosis, disseminated intravascular coagulation, and even pulmonary embolism, leading to a high death rate [1,2]. The prevalence of VTE is approximately 1 per 1000 pregnancies (0.025–0.1%) [3] and has increased since the coronavirus disease 2019 (COVID-19) pandemic [4,5,6]. Pregnancy is a well-known risk factor associated with hypercoagulation, increasing the risk of VTE four-to-five-fold compared to non-pregnant women [7,8].

Accordingly, D-dimer (DD) is considered an important test in ruling out a hypercoagulable state [9,10]. However, the concentration of DD increases with gestational age. A rapid rise in D-dimer levels at mid- and late pregnancy corresponds to a higher risk of developing adverse maternal and perinatal outcomes [11]. Recently, the soluble fibrin monomer complex (SFMC) has been considered a novel marker in predicting venous thromboses [12,13]. This agent is an early degenerated product of fibrin, which reflects a coagulation process. In contrast, this biomarker does not depend on the pregnancy trimester [14]. Since the rate of false positivity is lower, an elevated SFMC concentration could be a potential marker of thrombotic status in pregnancy rather than a thrombin–antithrombin complex and a D-dimer [15].

Although many practical guidelines have been widely applied, the Royal College of Obstetricians and Gynecologists (RCOG) guidelines remain a useful tool in rigorously assessing the potential risks for the development of VTE in puerperal women [16,17,18]. Nevertheless, substantive data concerning the role of FM in pregnancy remain limited in low- and middle-income countries. In Vietnam, the value of the SFMC has been documented in other fields, but no study has been published in pregnant women yet [19]. Thus, through this paper, we would like to address a novel finding relating the SFMC and D-dimer to VTE risk factors using the RCOG’s guidelines.

## 2. Materials and Methods

### 2.1. Study Population

This observational study was conducted at the Department of High-Risk Pregnancy, Tu Du Hospital, Vietnam from August 2023 to April 2024. Ethical approval was waived for this observational study at our institution. This study was registered at the following URL: https://osf.io/4g6sw (accessed on 21 April 2024).

**Figure 1:**
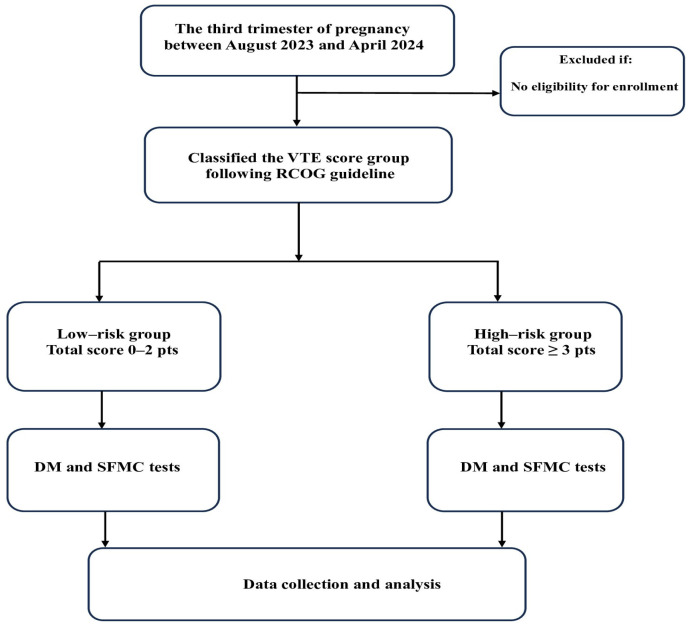
Study flowchart.

A study sample size calculation was not applicable in this study due to the availability of kit tests. The inclusion criteria led to the enrolment of 100 women in the third trimester of pregnancy who were evaluated for the risk of VTE according to the Royal College of Obstetricians and Gynecologists (RCOG) guidelines, with or without prior COVID-19 infection (Supplementary Table S1). The selected participants were classified either as low risk (risk score ≤ 2 pts) or high risk (risk score ≥ 3 pts) [20,21]. Exclusion criteria included the pregnant women delivered before collecting the blood sample patients, missing data, and pregnant women who refused to participate in the study (Figure 1). During the postpartum period, depending on the total VTE score classification of either intermediate risk or high risk of VTE, the participants received anticoagulant drugs using low-molecular-weight heparin (LMWH) standard prophylaxis (enoxaparin sodium 4000 UI/0.4 mL or enoxaparin sodium 6000 UI/0.6 mL following body weight (kg) for at least 1–6 weeks) combined with other mechanical preventions and were referred to a local expert according to hospital practical guidelines [22].

### 2.2. Study Tool

The blood sample was withdrawn following the patient’s consent. The blood was collected within 24 h of hospitalization and before induction of labor, vaginal birth, or cesarean section. STA-Liatest FM (100 test) and STA-Liatest D-Di Plus (100 test) were provided by MediGroup Asia company (Ho Chi Minh City, Vietnam). The tests were used with the analyzer machine labeled Sta-R Max (Sta-R Max, Diagnostica Stago, Asnières-sur-Seine, France).

### 2.3. Data Collection

The data were collected from the patient’s medical record. Categorical variables include sociodemographic characteristics, obstetric history, obstetric characteristics, materno-fetal outcomes, and risk factors for VTE before delivery. Regarding twin pregnancy or triplet pregnancy, the fetal weight, and amniotic fluid volume were collected with the greatest parameters, and the placenta location close to the internal os cervical was recorded. Continuous variables include maternal age (years), body mass index (kg/m^2^), platelet (1 × 10^9^/L), hemoglobin (g/L), red blood count (1 × 10^12^/L), prothrombin time (%), INR, APTT (s), TQ (s), D-dimer (µg/mL), soluble fibrin monomer complex (SFMC) (µg/mL), estimated blood loss (mL), and hospital length of stay (days).

### 2.4. Statistical Analysis

The data were entered into and analyzed by Statistical Package for the Social Sciences (SPSS) version 26.0 (IBM Corporation, New York, NY, USA). The data were presented as frequency (*n*), percentage (%), interquartile [Q1–Q3], and mean ± SD (min–max) depending on the distribution of data. Measurement information that was normally distributed was expressed as mean ± standard deviation (X ± SD), and the two groups were compared by the independent-samples *t*-test. Measurement information that was not normally distributed was expressed as median IQR [Q1–Q3], and the independent-samples Mann–Whitney U test was used to compare between two groups. Regarding categorical variables, the Chi-square test or Fisher’s Exact test was used, depending on the frequencies of data in each table cell.

Spearman and Pearson’s correlations (depending on the distribution of data) were used to determine the correlation coefficient (r) between gestational age (weeks) and SFMC and DD concentration, which ranged from −1 to +1, indicating a negative and positive correlation, respectively. An |r| value > 0.8 indicated a strong correlation, 0.4 ≤ |r| < 0.8 indicated a moderate correlation, and |r| < 0.4 indicated a weak correlation. The *p*-value (two-tailed) was considered to be statistically significant.

## 3. Results

In 100 pregnant women meeting the inclusion criteria, the present study found a significant difference concerning maternal age, BMI, gestational age, and fetal weight between the low-risk and high-risk groups for VTE before delivery (Table 1 and Table 2). No cases were recorded with a positive COVID-19 infection at the time of the study.

Table 3 shows no difference between the two groups regarding the parameters of the coagulation profile and serum finding tests including FM and DD. However, the concentration of tests seems likely to increase in the subgroup with preeclampsia, in vitro fertility (IVF), and multipregnancy (Table 4).

**Figure 2:**
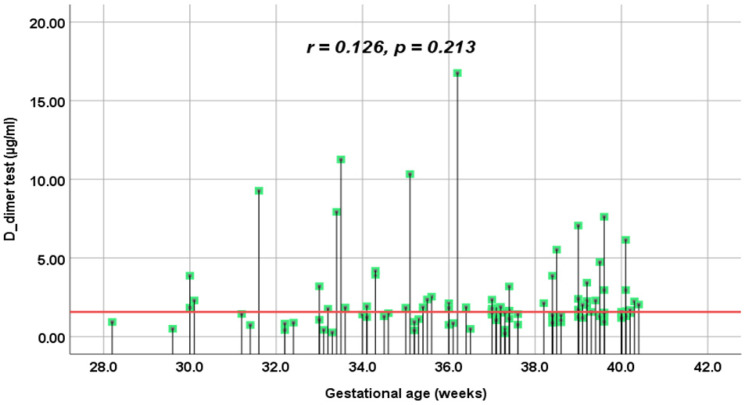
Correlation between D-dimer test (µg/mL) and gestational age (weeks) in the present study.

**Figure 3:**
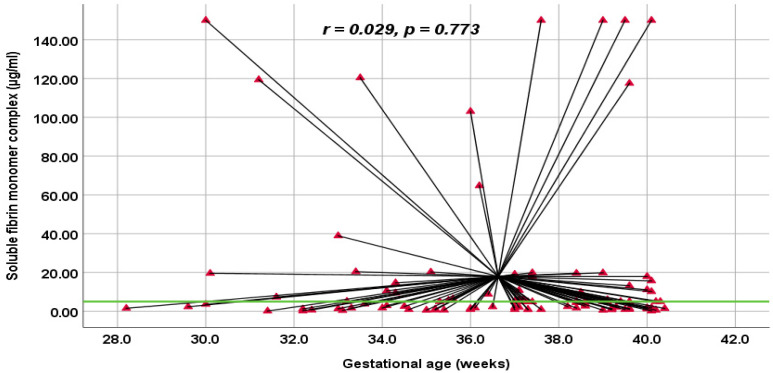
Correlation between soluble fibrin monomer complex test (µg/mL) and gestational age (weeks) in the present study.

**Figure 4:**
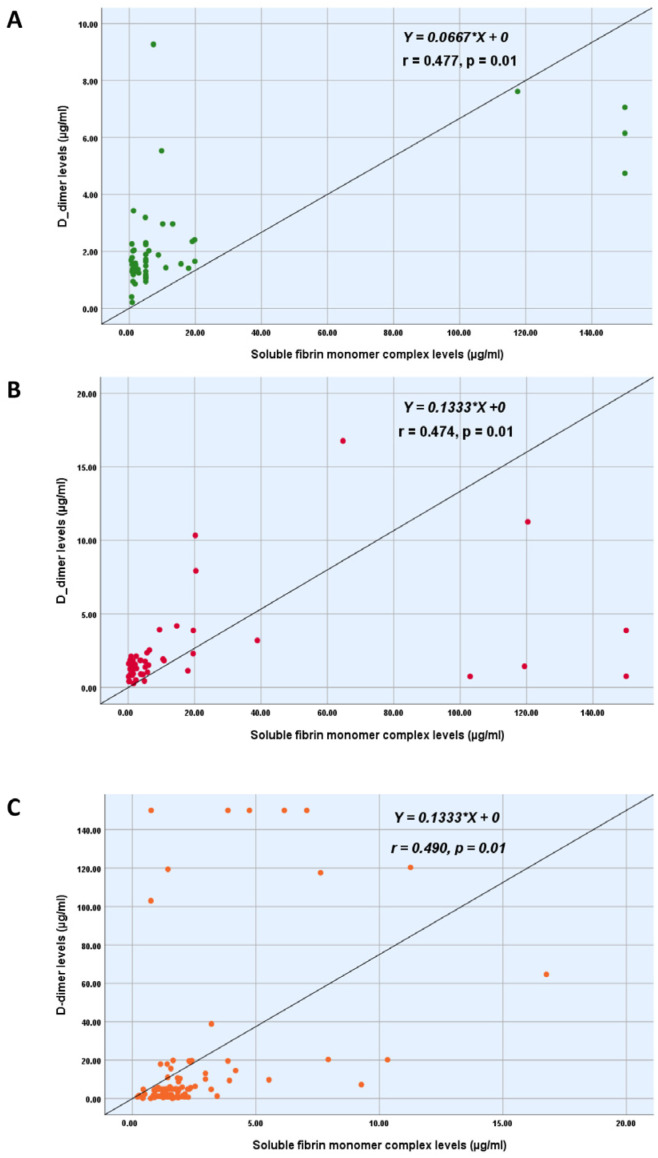
Correlation between D-dimer levels (µg/mL) and soluble fibrin monomer complex levels (µg/mL) in low-risk group (**A**), high-risk group (**B**), and in the overall study population (**C**).

Our study found the correlation between DD and gestational age is higher than that of FM (Figure 2 and Figure 3). However, both correlation results are weak and not statistically significant; *p* > 0.05. Moreover, using Spearman’s correlation coefficient, the present study revealed significantly a moderate positive correlation between the DD levels (µg/mL) and SFMC levels (µg/mL); *p* = 0.01 (Figure 4).

**Figure 5:**
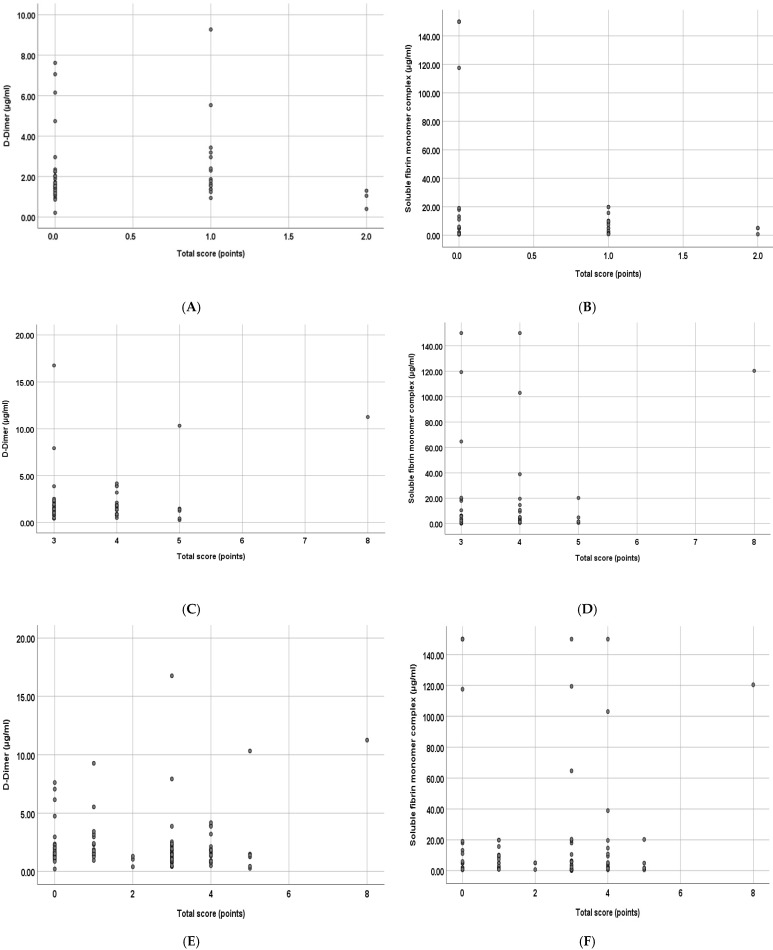
The correlation between VTE score (points) and the concentration of DD (μg/mL) in low-risk group (**A**), in high-risk group (**C**), and in the overall study (**E**). Similarly, the correlation between VTE score (points) and SFMC (μg/mL) in low-risk group (**B**), in high-risk group (**D**), and in the overall study (**F**).

Regarding the VTE score and the concentration of DD and SFMC, the present study found no significant correlation between fibrin-linked markers and the VTE score in low-risk group, in the high-risk group, and in the overall study, as shown in Table 5 and Figure 5.

Table 6 reveals the cesarean rate and hospital length of stay (days) in the high-risk group are greater than the low-risk group for VTE. The principal reason relating to the longer length of hospital stay is subcutaneous incision infection. In this study, none of the cases experienced thrombotic events, whether antepartum or postpartum.

## 4. Discussion

In a total of one hundred pregnant women, the median D-dimer (DD) and soluble fibrin monomer complex (SFMC) were 1.57 (µg/mL) (percentile 25th–75th: 1.15–2.29) and 4.94 (µg/mL) (percentile 25th–75th: 1.32–10.64), respectively. In the findings of Siennicka et al., the DD reference value ranges among pregnant women included 167–721 ng/mL (in the first trimester), 298–1653 ng/mL (in the second trimester), and 483–2256 ng/mL (in the third trimester) [23]. Onishi et al. showed the median SFMC in late pregnancy was 3.95 mg/L (2.74–5.16 mg/L) [15]. According to Kristoffersen et al., the median fibrin monomer concentration in pregnant women compared to non-pregnant women was 6.2 mg/L (percentiles 2.5th–97.5th: 3.7–10.8 mg/L) and 4.8 mg/L (3.6–8.2 mg/L) (*p* < 0.01) [14].

In our study, using the Royal College of Obstetricians and Gynecologists (RCOG) guideline assessment score of fewer than 2 points being considered a low-risk group and greater than 3 points a high-risk group, the study found no significant difference between the two groups concerning the SFMC and D-dimer (DD) level. Meanwhile, Iwamoto et al. have demonstrated that DD and SFMC concentrations were significantly higher in the high-risk group than in the low-risk group (DD 4.5 vs. 2.6 μg/mL, *p* = 0.008; SFMC 14.6 vs. 3.4 μg/mL, *p* < 0.001) [20].

Utilizing the three-point high-risk group concerning cumulative factors such as preeclampsia, multipregnancy, and having received assisted reproductive technologies, compared to the three-point high-risk group concerning several factors including maternal age more than 35 years old, parity greater than 3, and obesity, our study recognized that the concentration of SFMC and DD seems likely higher. However, the participants in these groups remain limited. As we know, the data used for building the RCOG risk assessment model were mostly obtained from Western countries [16]. According to the findings of Li et al., the RCOG risk assessment model was not a useful tool in predicting postpartum VTE. In high-risk patients, when combined with other biomarkers, this tool may be efficient [21]. In line with Grossman et al., the value of fibrin-linked markers for the exclusion of VTE in pregnancy could be better by adjusting for the maternal and obstetric characteristics of the patients [24]. Similarly, Siennicka et al. revealed that gestational diabetes and nicotinism as paramount factors ought to be taken into consideration for the risk of VTE in pregnancy [23].

The soluble fibrin monomer complex concentration is considerably stable during pregnancy; it is slightly higher than in non-pregnant women [14]. In accordance with Onishi et al., the variabilities in FM concentrations during normal pregnancy are minimal compared with other hemostatic markers [15]. According to the VTE score and fibrin-related markers, the present study found no significant correlation. Regarding the gestational age (GA), using Spearman’s correlation coefficient “r”, our study found no correlation between the GA and the level of SFMC and DD. This finding may be limited due to the small sample size and the fact that the pregnant women were included with a GA beyond 28 weeks of gestation. In line with the findings of Iwamoto et al., the FM concentration did not depend on the GA, but the DD concentration did [20]. Accordingly, Grossman et al. also found that DD varied following the gestational age rather than SFMC [24]. Nevertheless, we found a moderate positive correlation between D-dimer levels and soluble fibrin monomer complex levels in the present study.

Understandably, since the present study classified the participants into low-risk and high-risk groups, some materno-fetal characteristics and perinatal outcomes were significantly different. The high-risk group is associated with a higher rate of maternal age greater than 35 years old, obesity, lower birthweight, smaller gestational age, and longer length of hospital stay due to postpartum infection. At our center, all the pregnant women with high-risk scores of greater than 4 were indicated with thrombotic prophylaxis for at least 7 days. Overall, the study found no thrombotic cases during the hospital stay. Meanwhile, Grouzi et al. have found that 12 out of 742 women (1.6%) were involved in thrombotic events during the peripartum course [25].

### Strengths and Limitations

To the best of our knowledge, this study is presumably the first report in Vietnam concerning the RCOG score assessment for evaluating the risk of VTE before delivery. Additionally, our hospital is a tertiary referral center dealing with, principally, maternity health care; thus, pregnant women with a high VTE risk could be collected in a short time.

However, since our hospital is a specialized center, thromboembolic events are rarely recorded. Normally, hyper-coagulable patients are managed at general hospitals with a multidisciplinary team; thus, we missed this significant group. Therefore, the value of the SFMC and DD tests in diagnosing VTE was not studied. Moreover, the study sample size remains limited. In the future, we would like to investigate the value of SFMC compared to DD implementation in pregnant women with and without thromboembolic events.

## 5. Conclusions

In summary, our study found no significant difference between the low-risk and high-risk prenatal VTE regarding the soluble fibrin monomer complex (SFMC) and D-dimer (DD) concentration. In practice, these tests may be individualized and used among pregnant women with higher scores of VTE risk during antenatal care. Further data are required to strengthen this finding and optimize the routine indication of tests for healthcare professionals, especially in low-resource settings.

## Figures and Tables

**Table 1:** Baseline characteristics of the study population.

Characteristics		Group of VTE Risk		Total (N = 100)	*p*-Value
		Low-Risk (N = 50)	High-Risk (N = 50)		
Maternal age (years)		30.92 ± 0.80 (16–42)	36.42 ± 0.77 (22–56)	33.67 ± 0.62 (16–56)	<0.0001 *
Body mass index (kg/m^2^)		26.04 ± 0.48 (18.29–34.24)	34.45 ± 5.67 (20.02–36.98)	26.83 ± 0.50 (18.29–36.98)	0.025 *
Occupation	Houseworker	20 (48.8)	21 (51.2)	41 (100.0)	NA
	Worker	7 (36.8)	12 (63.2)	19 (100.0)	
	Farmer	0 (0.0)	2 (100.0)	2 (100.0)	
	Teacher/staff	17 (54.8)	14 (45.2)	31 (100.0)	
	Others	6 (85.7)	1 (14.3)	7 (100.0)	
Demographic region	HCM City	9 (60.0)	6 (40.0)	15 (100.0)	0.401 ^†^
	Others	41 (48.2)	44 (51.8)	85 (100.0)	
Gravida (times)		2.32 ± 1.38 (1–6)	2.80 ± 1.64 (1–7)	2.56 ± 1.53 (1–7)	0.116 *
Parity (times)	<3	46 (51.1)	44 (48.9)	90 (100.0)	0.505 ^†^
	≥3	4 (40.0)	6 (60.0)	10 (100.0)	
Miscarriage (times)	<3	49 (51.0)	47 (49.0)	96 (100.0)	0.617 ^††^
	≥3	1 (25.0)	3 (75.0)	4 (100.0)	
Cesarean section scar (times)	0	40 (51.9)	37 (48.1)	77 (100.0)	0.476 ^†^
	≥1	10 (43.5)	13 (56.5)	23 (100.0)	
Gestational age (weeks)		38.38 ± 0.26 (31.6–40.4)	34.88 ± 0.40 (28.2–40.1)	36.63 ± 0.30 (28.2–40.4)	<0.0001 *
History of disease	Hypertension	1 (16.7)	5 (83.3)	6 (100.0)	NA
	GDM	1 (33.3)	2 (66.7)	3 (100.0)	
	Systematic erythematous lupus	0 (0.0)	6 (100.0)	6 (100.0)	
	Nephrotic syndrome	0 (0.0)	2 (100.0)	2 (100.0)	
	Renal dysfunction	0 (0.0)	3 (100.0)	3 (100.0)	
	Hepatic dysfunction	0 (0.0)	1 (100.0)	1 (100.0)	
	Hypothyroidism	1 (33.3)	2 (66.7)	3 (100.0)	
	Hyperthyroidism	1 (25.0)	3 (75.0)	4 (100.0)	
	Other diseases	5 (45.45) ^a^	6 (54.55) ^b^	11 (100.0)	
Surgical history	Yes	13 (52.0)	12 (48.0)	25 (100.0)	0.817 ^†^
	No	37 (49.3)	38 (50.7)	75 (100.0)	

Data are presented as *n* (%), median [Q1–Q3], range (min–max), and mean (min–max). * Independent-t test (2-tailed). ^†^ Chi-square test. ^††^ Fisher’s Exact test. ^a^ Asthma (*n* = 1), chronic hepatitis B (*n* = 3), thyroid nodules (*n* = 1). ^b^ Asthma (*n* = 1), nephrotitis (*n* = 1), brain cancer surgery (*n* = 1), bicuspid valve regurgitation (*n* = 1), ventricular septal defect (*n* = 1), thyroid cancer (*n* = 1). HCM: Ho Chi Minh, GDM: gestational diabetes mellitus, VTE: venous thromboembolism.

**Table 2:** Materno-fetal characteristics.

Characteristics		Group of VTE Risk		Total	*p*-Value
		Low-Risk (N = 50)	High-Risk (N = 50)		
Number of fetuses	Singleton	49 (57.6)	36 (42.4)	85 (100.0)	NA
	Twin	0 (0.0)	13 (100.0)	13 (100.0)	
	Triplet	1 (50.0)	1 (50.0)	2 (100.0)	
Fetal weight (gram)	Mean ± SD (min–max)	3052.04 ± 435.16 (1470–3760)	2270.18 ± 614.45 (1150–3600)	2661.11 ± 65.95 (1150–3760)	<0.0001 *
Amniotic fluid volume (mL)	Median IQR [1–3]	10.50 [7.00–14.00] (3.00–28.00)	11.00 [8.00–19.00] (3.00–68.00)	11.00 [7.20–17.00] (3.00–68.00)	0.483 **
Largest amniotic fluid poche (cm)	Mean ± SD	4.19 ± 1.52	5.06 ± 2.92	4.71 ± 0.31 (1.1–20.0)	0.117 *
Intact amniotic membrane rupture	Yes	45 (47.9)	49 (52.1)	94 (100.0)	NA
	No	5 (83.3)	1 (16.7)	6 (100.0)	
Accompanied disease	Gestational hypertension	0 (0.0)	1 (100.0)	1 (100.0)	NA
	Non-severe preeclampsia	2 (22.2)	7 (77.8)	9 (100.0)	
	Severe preeclampsia	2 (5.7)	33 (94.3)	35 (100.0)	
	Non-medical GDM	6 (54.5)	5 (45.5)	11 (100.0)	
	Insulin-treated GDM	0 (0.0)	4 (100.0)	4 (100.0)	
	Other diseases	1 (12.5)	7 (87.5)	8 (100.0)	
Risk factors for VTE before delivery	Non-surgical previous thrombus	0 (0.0)	1 (100.0)	1 (100.0)	NA
	Surgical previous thrombus	0 (0.0)	0 (0.0)	0 (0.0)	
	High-risk embolism	0 (0.0)	1 (100.0)	1 (100.0)	
	Severe medical disease	0 (0.0)	15 (100.0)	15 (100.0)	
	Familial history of thrombus	0 (0.0)	0 (0.0)	0 (0.0)	
	Low-risk thromboembolism	0 (0.0)	0 (0.0)	0 (0.0)	
	Maternal age greater than 35 years old	9 (19.6)	37 (80.4)	46 (100.0)	
	Obesity	0 (0.0)	7 (100.0)	7 (100.0)	
	Parity greater than 3	8 (25.0)	24 (75.0)	32 (100.0)	
	Smoking	0 (0.0)	1 (100.0)	1 (100.0)	
	Gross varicose of lower limbs	0 (0.0)	0 (0.0)	0 (0.0)	
	Preeclampsia in pregnancy	4 (9.8)	37 (90.2)	41 (100.0)	
	In vitro fertility	1 (7.1)	13 (92.9)	14 (100.0)	
	Multipregnancy	1 (5.9)	16 (100.0)	17 (100.0)	
	Other risk factors	0 (0.0)	2 (100.0) ^†^	2 (100.0)	
Total scores	Median (min–max)	0 [0–1] (0–2)	4 [3–4] (3–8)	2.5 [0–4] (0–8)	<0.0001 **

Data are presented as *n* (%), median [Q1–Q3], range (min–max), and mean ± standard deviation (min–max). * Independent-*t* test (2-tailed) ** Nonparametric test (independent-samples Mann–Whitney U test). ^†^ Ovarian hyperstimulation, varicose veins on the legs. GDM: gestational diabetes mellitus, VTE: venous thromboembolism.

**Table 3:** The serum laboratory findings of the study population.

Parameters		Group of VTE Risk		Total	*p*-Value
		Low-Risk (N = 50)	High-Risk (N = 50)		
Total blood cell count	Platelet (10^9^/L)	249.72 ± 7.77 (147–456)	235.64 ± 9.52 (81–392)	242.68 ± 6.15 (81–456)	0.255 *
	Hemoglobin (g/L)	122.56 ± 1.49 (95–141)	116.85 ± 4.85 (11–151)	119.70 ± 2.54 (11–151)	0.265 *
	Red blood count (10^12^/L)	4.54 ± 0.22 (3.45–13.42)	4.43 ± 0.14 (9.70–221.40)	4.48 ± 0.13 (2.49–13.42)	0.677 *
Coagulation profile	PT (%)	104.54 ± 1.28 (88.0–129.4)	105.32 ± 2.58 (12.7–142.5)	104.93 ± 1.43 (12.7–142.5)	0.786 *
	INR	0.98 ± 0.01 (0.88–1.09)	0.95 ± 0.13 (0.54–1.13)	0.97 ± 0.01 (0.54–1.13)	0.072 *
	APTT (s)	29.89 ± 0.64 (3.60–34.80)	30.96 ± 0.47 (22.10 ± 36.50)	30.43 ±0.40 (3.60–36.50)	0.179 *
	TQ (s)	12.70 (9.40–14.00) [12.40–13.10]	12.50 (11.90–13.10) [11.90–13.10]	12.6 (12.1–13.1) [8.90–14.50]	0.444
Study tests	DD (µg/mL)	1.61 (0.21–9.27) [1.30–2.30]	1.51 (0.27–16.76) [0.91–2.13]	1.57 (0.21–16.76) [1.15–2.29]	0.282 **
	SFMC (µg/mL)	5.00 (0.54–150.00) [1.36–9.78]	3.74 (0.10–150.00) [1.28–14.63]	4.94 (0.10–150.00) [1.32–10.64]	0.882 **

Data are presented as *n* (%), median [Q1–Q3], range (min–max), and mean ± standard deviation (min–max). * Independent-t test (2-tailed) ** Nonparametric test (independent-samples Mann–Whitney U test). Fibrinogen in the total study (N = 10): 390.50 (g/L) [4.94–526.20] (3.39–742.00), in high-risk group (N = 8): 440.50 [191.54–535.35] (4.94–742.00), and in low-risk group (N = 2): 3.50 [3.39–3.60] (3.39–3.60). APTT: activated partial thromboplastin time, DD: D-dimer, PT: prothrombin time, TQ: time quick, INR: international normalized ratio, SFMC: soluble fibrin monomer complex, VTE: venous thromboembolism.

**Table 4:** The value of DD and SFMC in specific groups.

Finding Tests	Group 1 (N = 10)	Group 2 (N = 4)
DD (µg/mL)	3.57 [2.06–8.76] (1.77–16.76)	0.91 [0.69–1.16] (0.49–1.39)
SFMC (µg/mL)	12.03 [5.10–38.9] (1.44–120.37)	1.92 [1.07–3.72] (0.62–5.12)

Data are presented as median [Q1–Q3] and range (min–max). DD: D-dimer, SFMC: soluble fibrin monomer complex. Group 1: Preeclampsia, assisted reproductive technology, and multipregnancy. Group 2: Maternal age greater than 35 years old, obesity, and parity greater than 3. Regarding the case of ovarian stimulation syndrome (DD: 11.26 (µg/mL), and SFMC: 120.37 (µg/mL)).

**Table 5:** Correlation between VTE score (points) and DD/SFMC (µg/mL) in the present study.

VTE group	Low-Risk		High-Risk		Overall Study	
	DM	SFMC	DM	SFMC	DM	SFMC
Spearman’s coefficient (r)	−0.095	−0.007	0.004	0.057	−0.002	−0.031
*p*-value (2-tailed)	0.347	0.943	0.980	0.64	0.989	0.832

**Table 6:** Materno-fetal outcomes.

Outcomes		Group of VTE Risk		Total (N = 100)	*p*-Value
		Low-Risk (N = 50)	High-Risk (N = 50)		
Birth method	Cesarean section	26 (37.7)	43 (62.3)	69 (100.0)	0.0001 ^†^
	Vaginal birth ^a^	24 (77.4)	7 (22.6)	31 (100.0)	
Induction of labor	No required	30 (50.0)	30 (50.0)	60 (100.0)	NA
	Foley balloon catheter ^b^	20 (52.6)	18 (47.4)	38 (100.0)	
	Dinoprostone ^b^	0 (0.0)	2 (100.0)	2 (100.0)	
Indication of cesarean	Failed IOL	8 (42.1)	11 (57.9)	19 (100.0)	NA
	Non-reassuring fetal CTG	5 (45.5)	6 (54.5)	11 (100.0)	
	Placenta previa	7 (87.5)	1 (12.5)	8 (100.0)	
	Twin pregnancy underwent IVF	0 (0.0)	11 (100.0)	11 (100.0)	
	CS scar ≥ 2 times	1 (12.5)	7 (87.5)	8 (100.0)	
	Other	3 (30.0)	7 (100.0)	10 (100.0)	
Total blood during delivery (mL)	<500	44 (50.6)	43 (49.4)	87 (100.0)	0.766 ^†^
	≥500	6 (46.2)	7 (53.8)	13 (100.0)	
Intraoperative EBL (mL)	Median ± SD (min–max)	300 [200–400] (200–1400)	300 [200–300] (200–700)	300 [200–300] (200–1400)	0.693 **
Hospital length of stay (days)	Median ± SD (min–max)	6 [5–8] (3–27)	8 [7–9] (3–30)	7 [5.5–9] (3–30)	0.004 **
Postoperative time duration (days)	Median ± SD (min–max)	5 [3–5] (3–25)	6 [5–7] (3–17)	5 [4.5–6] (3–25)	<0.0001 **
Postpartum infection	No	49 (51.6)	46 (48.4)	95 (100.0)	0.117 ^††^
	Yes	0 (0.0)	4 (100.0)	4 (100.0) ^c^	
Apgar score at 1 min (points)	≤3	0 (0.0)	1 (100.0)	1 (100.0)	1.0 ^††^
	>3	50 (50.5)	49 (49.5)	99 (100.0)	
Apgar score at 5 min (points)	<7	1 (14.3)	6 (85.7)	7 (100.0)	0.112 ^††^
	≥7	49 (52.7)	44 (47.3)	93 (100.0)	
Mode of neonatal care	Skin-to-skin	32 (91.4)	3 (8.6)	35 (100.0)	NA
	Neonatal unit admission <24 h	17 (30.4)	39 (69.6)	55 (100.0)	
	NICU admission	1 (11.1)	8 (88.9)	9 (100.0)	

Data are presented as *n* (%), median [Q1–Q3], range (min–max), and mean ± standard deviation (min–max). ** Nonparametric test (independent-samples Mann–Whitney U test) ^†^ Chi-square test ^††^ Fisher’s Exact test. ^a^ Assisted instrumental vaginal birth (*n* = 4, 2 cases in each group). ^b^ IOL with/without oxytocin infusion. No blood transfusion was required in the present data. ^c^ Postpartum infection included 4 cases with surgical site infection which responded to antibiotic therapy. One triplet-pregnant woman was transferred to another tertiary general hospital (C.R. Hospital) at 33 weeks of gestation due to a gastric ulcer since the data were missed for postpartum infection. No cases were required for blood transfusion and hysterectomy in this study. CTG: cardiotocography, CS: cesarean section, IOL: induction of labor, IVF: in vitro fertility, NICU: neonatal intensive care unit, VTE: venous thromboembolism.

## Data Availability

The datasets used and/or analyzed during the current study are available from the corresponding author upon reasonable request.

## Supplementary Materials

The following supporting information can be downloaded at: https://www.mdpi.com/article/10.3390/jcm14051399/s1, Supplementary Table S1. The RCOG risk assessment model for VTE.

## Abbreviations

C-section: cesarean section, DD: D-dimer, SFMC: soluble fibrin monomer complex, RCOG: the Royal College of Obstetricians and Gynecologists, VTE: venous thromboembolism.
