# Supplementary material for: Soluble Fibrin Monomer Complex and D-Dimer Concentrations Between Patients at Low and High Risk of Venous Thromboembolism Before Delivery According to RCOG Score Assessment: An Observational Study Among 100 Third-Trimester Vietnamese Pregnancies
Source: J Clin Med. 2025 Feb 20;14(5):1399. doi: 10.3390/jcm14051399 (PMC11900025; doi:10.3390/jcm14051399)
Supplement: Supplementary file 1 [file jcm-14-01399-s001.zip › jcm-3237390-supplementary.pdf]

Supplementary Table S1. The RCOG risk assessment model for VTE.

Risk factors for VTE		Score
**Pre-existing risk factors**		
Previous VTE (except a single event related to major surgery)		4
Previous VTE provoked by major surgery		3
Known high-risk thrombophilia		3
Medical comorbidities (e.g., cancer, heart failure; active systemic lupus erythematosus, Inflammatory polyarthropathy or inflammatory bowel disease; nephrotic syndrome; type I diabetes mellitus with nephropathy; sickle cell disease; current intravenous drug user)		3
Family history of unprovoked or estrogen-related VTE in first-degree relative		1
Known low-risk thrombophilia (no VTE)		1
Age (>35 years)		1
Obesity	BMI ≥ 30	1
	BMI ≥ 40	2
Parity ≥ 3		1
Smoker		1
Gross varicose veins		1
**Obstetric risk factors**		
Pre-eclampsia in current pregnancy		1
ART/IVF (antenatal only)		1
Multiple pregnancy		1
Cesarean section in labor		2
Elective cesarean section		1
Mid-cavity or rotational operative delivery		1
Prolonged labor (>24 h)		1
PPH (>1 L or transfusion)		1
Preterm birth < 37+0 weeks in current pregnancy		1
Stillbirth in current pregnancy		1

**Transient risk factors**	
Any surgical procedure in pregnancy or puerperium except immediate repair of the perineum, e.g., appendicectomy, postpartum sterilization	3
Hyperemesis	3
OHSS (first trimester only)	4
Current systemic infection	1
Immobility, dehydration	1
VTE – venous thromboembolism; BMI – body mass index; ART – assisted reproductive technology; IVF – in vitro fertilization; PPH – postpartum hemorrhage. ART/IVF is regarded as a risk factor for antenatal VTE, and ovarian hyperstimulation syndrome (OHSS) is regarded as a risk factor for VTE only in the first trimester.	
